# Transverse maxillary dimensions and upper airway morphology in mouth- and nasal-breathing children aged 10–12 years: A CBCT-based study

**DOI:** 10.1016/j.jobcr.2025.07.009

**Published:** 2025-07-22

**Authors:** Rani Satiti, Hendri Susanto, Anrizandy Narwidina

**Affiliations:** a Master Program in Clinical Dentistry, Faculty of Dentistry, Universitas Gadjah Mada, Yogyakarta, Indonesia; b Department of Oral Medicine, Faculty of Dentistry, Universitas Gadjah Mada, Yogyakarta, Indonesia; c Department of Pediatric Dentistry, Faculty of Dentistry, Universitas Gadjah Mada, Yogyakarta, Indonesia

**Keywords:** Mouth breathing, CBCT, Maxillary width, Upper airway, Pharyngeal airway

## Abstract

**Introduction:**

Mouth breathing (MB) is a dysfunctional respiratory pattern that may affect craniofacial development by altering maxillary arch width and upper pharyngeal airway morphology. Early identification is critical to prevent long-term dentofacial and airway complications. This study aimed to compare maxillary arch width and upper airway morphology between mouth- and nasal-breathing children aged 10–12 years using Cone Beam Computed Tomography (CBCT).

**Materials and methods:**

In this cross-sectional study, 30 children (15 mouth breathers and 15 nasal breathers) underwent CBCT imaging. Transverse maxillary arch dimensions were measured at four points: maxillary width at molars (MWM), intermolar width (IMW), maxillary width at canines (MWC), and intercanine width (ICW). Upper airway morphology was assessed using volumetric (nasopharyngeal volume [NPV], oropharyngeal volume [OPV]) and cross-sectional area (nasopharyngeal area [NPA], oropharyngeal area [OPA]) measurements. Independent *t*-tests were used to compare group differences with 95 % confidence level.

**Results:**

The MB group showed significantly reduced maxillary arch widths (MWM, IMW, MWC, ICW) and diminished upper airway volume and area (NPV, OPV, NPA, OPA) compared to nasal breathers (*p* < 0.001 for all parameters).

**Conclusion:**

Mouth breathing in school-aged children is associated with measurable reductions in maxillary arch width and upper pharyngeal airway dimensions.

## 1 Introduction

Mouth breathing (MB) is a commonly observed dysfunctional habit that can interfere with normal craniofacial growth and airway development. It alters orofacial soft tissue posture and may compromise both skeletal alignment and airway patency.^1^^,^^2^ In contrast, nasal breathing (NB) promotes optimal transverse development of the maxilla and contributes to airway stability by maintaining appropriate tongue posture.^3^ Children who primarily breathe through the mouth often exhibit compensatory changes such as open-mouth posture and low tongue position, which can narrow the upper jaw, contribute to posterior crossbite, and reduce the upper pharyngeal airway space.^4^^,^^5^

Beyond local structural effects, MB is associated with systemic consequences such as intermittent hypoxia, which may adversely impact cognitive and general health.^6^ Early and accurate diagnosis is essential, yet conventional assessments are often subjective. CBCT provides precise 3D imaging for evaluating both skeletal and airway structures, improving diagnostic precision and enabling timely interdisciplinary care.^7^

The pharynx, a critical conduit for respiration and a determinant of orofacial morphology, is particularly susceptible to functional narrowing in MB cases.^8^^,^^9^ While airway volume has been widely studied, recent evidence suggests that minimal cross-sectional area may be a more accurate predictor of airway resistance.^10^^,^^11^ Simultaneous evaluation of both volume and area via CBCT offers a more complete understanding of airway compromise.^12^

Despite growing international interest, region-specific data on MB remain scarce, particularly in Indonesia. No prior CBCT-based studies have examined its impact on maxillary width and pharyngeal airway morphology in Indonesian children, despite high prevalence estimates in urban populations.^13^ Ages 10–12 represent a critical window for interceptive interventions in craniofacial development.^14^ To address this gap, the present study evaluates differences in maxillary arch width and upper pharyngeal airway morphology between MB and NB children using CBCT. Findings aim to support early diagnosis and regionally tailored, interdisciplinary intervention.

## 2 Materials and methods

The study conducted at elementary schools in Kotagede District, Yogyakarta, Indonesia. This observational study employed a cross-sectional design with purposive sampling.

### 2.1 Inclusion and exclusion criteria

Ethical approval was obtained from the Institutional Review Board of Universitas Gadjah Mada and Dental Hospital, RSGM UGM Prof. Soedomo (No. 232/UN1/KEP/FKG-RSGM/EC/2024). Informed consent was provided by parents. Inclusion criteria were children aged 10–12 years with clinically diagnosed mouth breathing (MB), no systemic illnesses affecting airway patency, and no prior orthodontic treatment. Exclusion criteria included febrile conditions, allergic rhinitis, a history of tonsillectomy or adenoidectomy, and the presence of enlarged tonsils or palatal tissue, diagnosed via intraoral visual inspection.

During pubertal growth, lymphoid tissues such as the adenoids and palatine tonsils can enlarge to approximately 200 % of their adult size, potentially narrowing the airway and introducing bias in volumetric assessment. This enlargement is often more prominent in mouth-breathing children due to chronic inflammation and altered airflow dynamics. As such, age-matching and standardized breathing pattern classification are essential to reduce confounding and ensure valid comparisons. Diagnosis of MB was conducted using a multi-step approach: the Airway Index, clinical examinations (Quinn's Nasal Competency Test, Mallampati Classification, tonsillar grading, and Mirror Test), and validated parental questionnaires (with prior validation using Pearson correlation analysis in SPSS). Control subjects (nasal breathers) were identified using the same diagnostic protocol.

### 2.2 Sample size calculation

Sample size estimation was performed using G∗Power software for a two-tailed independent *t*-test. An effect size of 1.33 was derived based on an expected mean difference of 20 mm^3^ and a standard deviation of 15 mm^3^ for oropharyngeal volume. With α set at 0.05 and power at 80 %, the minimum required sample size was 12 participants per group. To enhance statistical reliability, 15 subjects were recruited in each group.

### 2.3 CBCT-based measurements of maxillary arch and pharyngeal airway

**Fig. 1:**
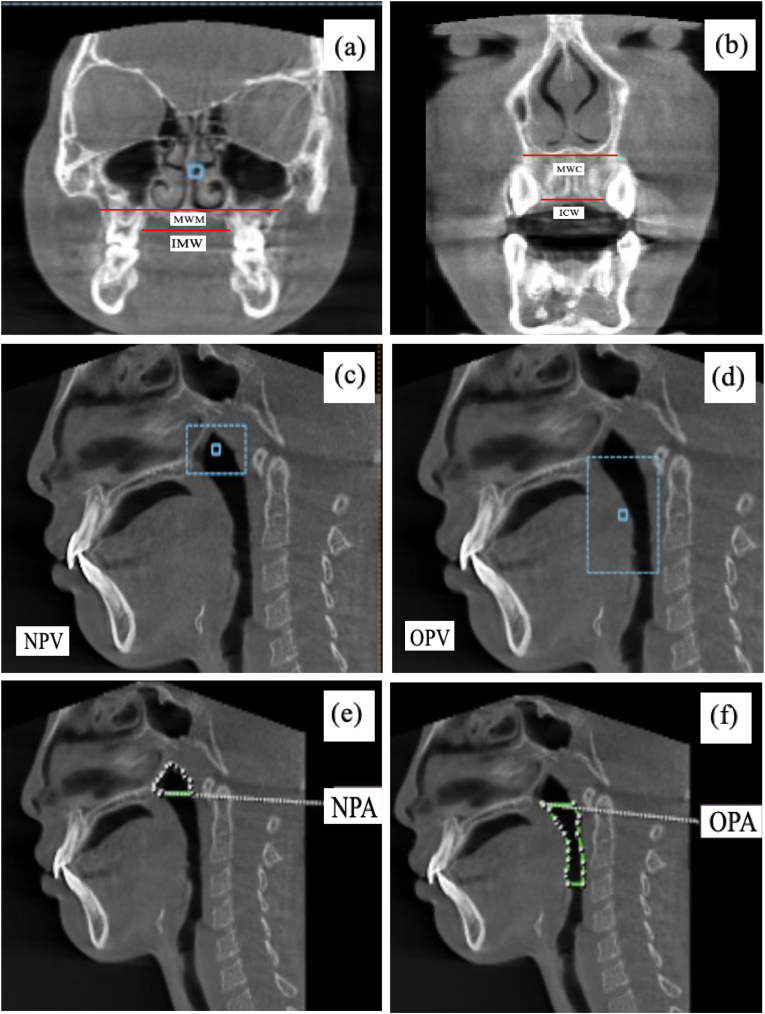
CBCT-based measurement methods: (a) transverse maxillary arch width of molar area; (b) transverse maxillary arch width of canine area; (c) nasopharyngeal airway volume; (d) oropharyngeal airway volume; (e) nasopharyngeal cross-sectional area; (f) oropharyngeal cross-sectional area.

Three-dimensional radiographic data were acquired using a standardized cone-beam computed tomography (CBCT) system (Orthopantomograph® OP300, Instrumentarium, KaVo Kerr, Finland) with settings of 90 kVp and 16 mA. All scans were evaluated using OnDemand3D® software (Cybermed Inc., Seoul, Korea). Head orientation was standardized using the Frankfort horizontal plane. During image acquisition, participants were instructed to remain motionless, breathe nasally, and maintain tongue-to-palate posture. Transverse maxillary dimensions were measured at four landmarks—MWM, IMW, MWC, and ICW—using linear distances between bilateral skeletal or dental points (Fig. 1a and b). Airway volumes were segmented into nasopharyngeal (NPV) and oropharyngeal (OPV) regions, measured along the Frankfort horizontal plane (Fig. 1c and d). Minimum cross-sectional areas of the nasopharynx (NPA) and oropharynx (OPA) were determined from axial slices at their narrowest points (Fig. 1e and f).^11^^,^^12^ All measurements were performed by a single calibrated examiner. Intra-examiner reliability was assessed by duplicate measurements on 10 randomly selected CBCT scans, yielding intraclass correlation coefficients (ICC) ranging from 0.87 to 0.93.

### 2.4 Statistical analysis

All quantitative data were analyzed using SPSS software, version 27 (IBM Corp., Armonk, NY, USA). Normal distribution of the data was evaluated using the Shapiro–Wilk test, while homogeneity of variances was assessed with Levene's test. Upon confirming that these assumptions were met, comparisons between mouth-breathing and nasal-breathing groups were conducted using independent samples t-tests, with statistical significance set at a 95 % confidence level.

## 3 Results

A total of 30 children were enrolled and equally allocated into two groups: 15 mouth breathers (MB) and 15 nasal breathers (NB). The mean age of participants was 11.2 ± 0.6 years. The MB group comprised 10 males and 5 females, while the NB group included 9 males and 6 females. Intra-examiner reliability was evaluated by repeating measurements in 10 randomly selected cases, yielding intraclass correlation coefficients (ICC) ranging from 0.87 to 0.93.

### 3.1 Maxillary dental arch width

**Fig. 2:**
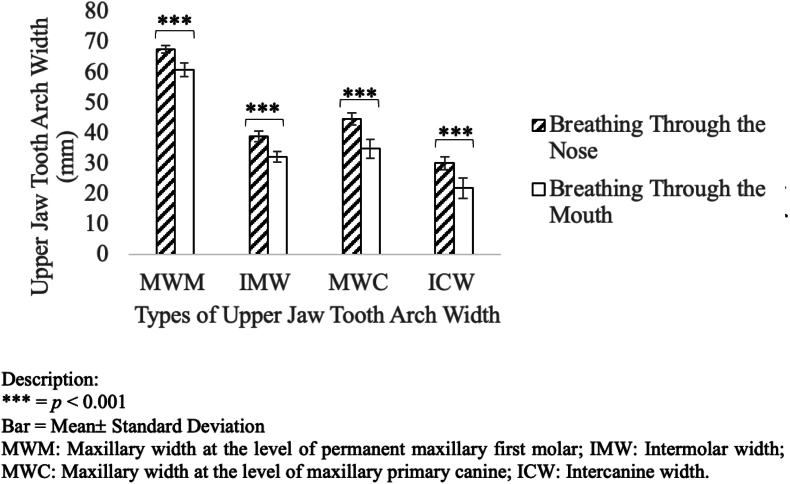
Mean maxillary arch widths in MB vs. NB children. All parameters showed statistically significant reductions in the MB group. Error bars represent SEM.

CBCT analysis revealed that children in the mouth breathing (MB) group exhibited significantly reduced transverse maxillary dimensions compared to nasal breathing (NB) controls (Table 1). All measured parameters—MWM, IMW, MWC, and ICW—were markedly narrower in MB subjects (*p* < 0.001), indicating constriction of the dental arch (Fig. 2). These findings may reflect disrupted orofacial muscular equilibrium and altered tongue posture commonly observed in chronic mouth breathers, with potential implications for malocclusion development and maxillary growth trajectory.

**Table 1:** Comparison of maxillary arch width between MB and NB groups.

Parameter	MB (Mean ± SD)	NB (Mean ± SD)	% Reduction	*p*-value
MWM (mm)	60.93 ± 2.25	67.66 ± 1.29	10.0 %	<0.001
IMW (mm)	32.35 ± 1.75	38.97 ± 1.77	17.0 %	<0.001
MWC (mm)	34.91 ± 3.11	44.70 ± 1.97	22.0 %	<0.001
ICW (mm)	21.99 ± 3.40	30.13 ± 2.16	27.0 %	<0.001

### 3.2 Upper airway volume

**Fig. 3:**
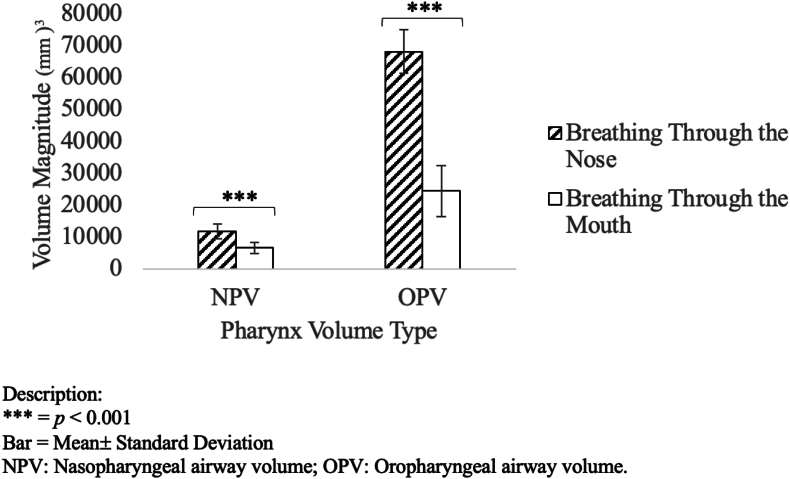
Nasopharyngeal and oropharyngeal airway volumes in MB and NB subjects.

Both nasopharyngeal and oropharyngeal volumes were significantly lower in the MB group (*p* < 0.001), suggesting compromised upper airway patency (Fig. 3). The nasopharyngeal airway volume (NPV) was reduced by 44 %, and the oropharyngeal airway volume (OPV) by 64 % (Table 2).

**Table 2:** Upper airway volume comparison.

Parameter	MB (Mean ± SD)	NB (Mean ± SD)	% Reduction	*p*-value
NPV (mm^3^)	6572.09 ± 1765.59	11697.48 ± 2241.29	44.0 %	<0.001
OPV (mm^3^)	24342.17 ± 8086.51	68005.19 ± 6850.71	64.0 %	<0.001

### 3.3 Upper airway area

**Fig. 4:**
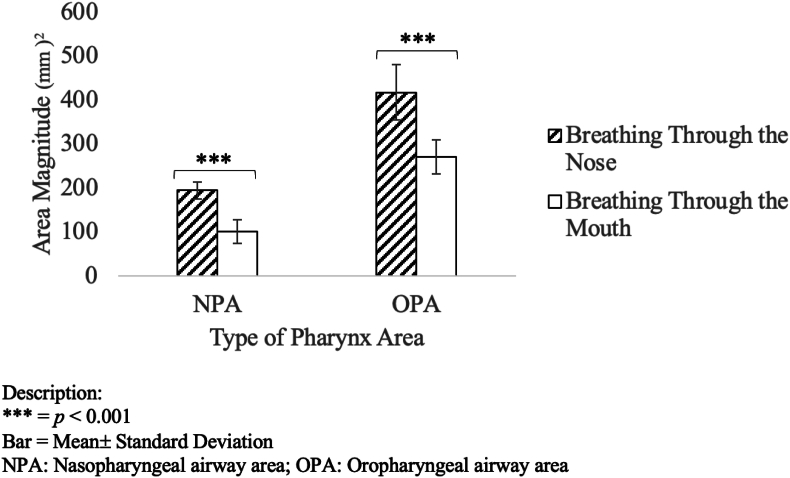
NPA and OPA were significantly reduced in MB subjects (*p* < 0.001). Error bars represent SEM.

Cross-sectional area measurements of the nasopharyngeal (NPA) and oropharyngeal (OPA) regions were significantly reduced in the MB group compared to nasal breathers, with mean reductions of 48 % and 35 %, respectively (*p* < 0.001; Table 3; Fig. 4). These findings suggest a potential narrowing of the upper airway lumen, which may contribute to functional airflow limitation and altered orofacial adaptation during growth.

**Table 3:** Upper airway area comparison.

Parameter	MB (Mean ± SD)	NB (Mean ± SD)	% Reduction	*p*-value
NPA (mm^2^)	100.92 ± 26.46	194.06 ± 19.68	48.0 %	<0.001
OPA (mm^2^)	269.82 ± 38.50	417.55 ± 62.47	35.0 %	<0.001

### 3.4 CBCT visualization

**Fig. 5:**
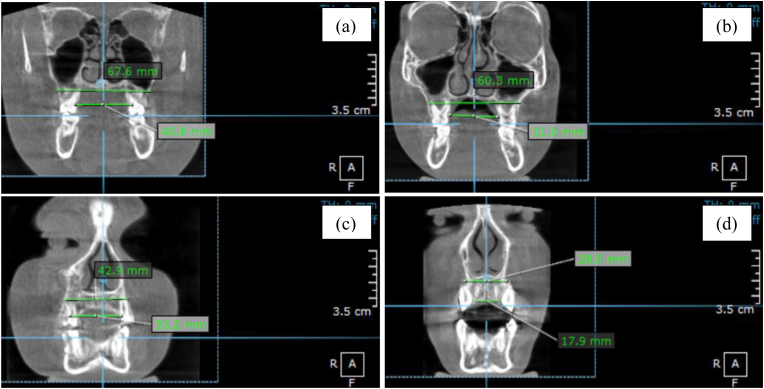
Representative CBCT images of maxillary arch width measurements in nasal-breathing (NB) and mouth-breathing (MB) children. (a, b) MWM and IMW in NB and MB groups, respectively; (c, d) MWC and ICW in NB and MB groups, respectively.

Three-dimensional CBCT reconstructions qualitatively confirmed the quantitative findings. Children in the mouth breathing (MB) group exhibited visible transverse maxillary constriction compared to nasal breathing (NB) peers. As shown in Fig. 5, all transverse dimensions—including MWM, IMW, MWC, and ICW—were reduced in the MB group, supporting the association between chronic oral breathing, and altered maxillary development.

**Fig. 6:**
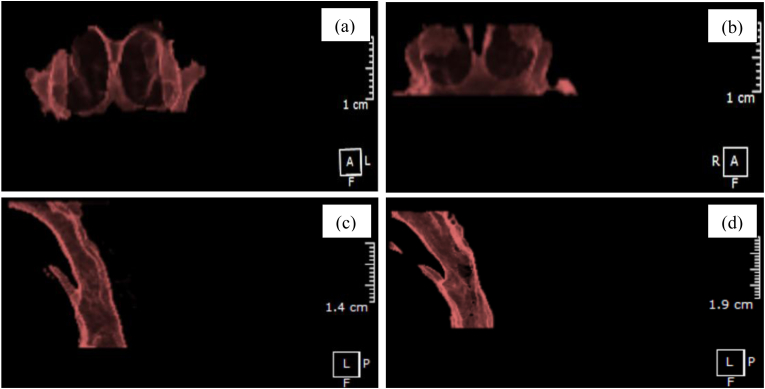
CBCT-based 3D reconstructions of nasopharyngeal (NPV) and oropharyngeal (OPV) volumes in nasal-breathing (NB) and mouth-breathing (MB) children. (a, b) NPV in NB and MB groups; (c, d) OPV in NB and MB groups.

Upper airway volume measurements are visualized in Fig. 6, which displays reconstructions of the nasopharyngeal (NPV) and oropharyngeal (OPV) compartments. Both volumetric parameters were substantially lower in MB subjects, reflecting a trend toward compromised airway patency that may accompany orofacial adaptations to dysfunctional breathing.

**Fig. 7:**
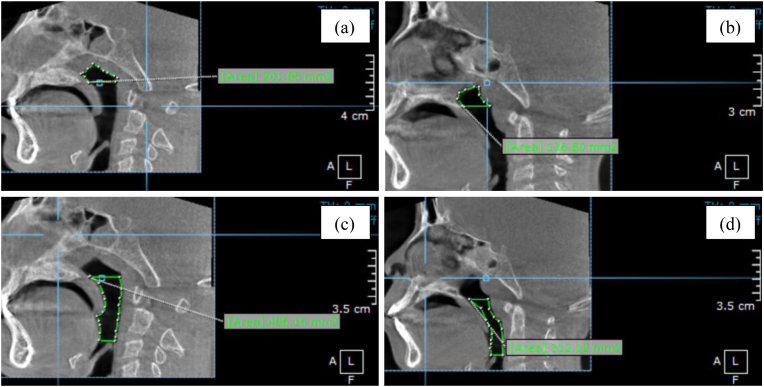
Axial CBCT images illustrating nasopharyngeal (NPA) and oropharyngeal (OPA) cross-sectional areas in NB and MB groups™. (a, b) Nasopharyngeal area (NPA) in nasal-breathing (NB) and mouth-breathing (MB) groups, respectively. (c, d) Oropharyngeal area (OPA) in NB and MB groups, respectively.

Fig. 7 presents axial CBCT images highlighting reduced nasopharyngeal (NPA) and oropharyngeal (OPA) airway areas in mouth-breathing (MB) children. These reductions support the role of CBCT in identifying airway narrowing associated with altered respiratory function during growth.

## 4 Discussion

This CBCT-based study confirms that mouth breathing (MB) in children aged 10–12 years is significantly associated with reduced maxillary arch width and diminished upper pharyngeal airway volume and area. These findings support the premise that altered respiratory patterns during key phases of craniofacial development may negatively impact skeletal morphology and upper airway structure. Notably, the hypopharyngeal region was not evaluated in this study, as reliable segmentation and landmark identification within this area remain technically challenging in pediatric CBCT due to anatomical variability and limited field-of-view.

A higher prevalence of MB was observed among male participants, which aligns with evidence suggesting sex-related behavioural and physiological factors, such as greater physical activity, stress levels, and oral habit prevalence.^15^, ^16^, ^17^ MB is influenced by both anatomical and behavioural components. The predominance of MB in children aged 10–11 may reflect a developmental window characterized by mixed dentition and early pubertal growth, during which craniofacial structures remain highly responsive to functional stimuli.^15^, ^16^, ^17^, ^18^

Occlusal analysis showed all nasal breathers had Class I molar relationships, while mouth breathers exhibited varied patterns, including Class II Division 1. This indicates that low tongue posture and muscle imbalance may disrupt orofacial equilibrium, contributing to maxillary constriction and mandibular retrusion.^19^ The relationship between mouth breathing and Class II patterns may be bidirectional, as mandibular retrusion and weak lip seal can impair nasal airflow, promoting oral breathing.^20^^,^^21^ Mallampati scores of 1–2 and CBCT evidence of narrowed but non-obstructive airways suggest mouth breathing is often functional rather than structural.^1^^,^^22^^,^^23^ These findings highlight the importance of assessing skeletal, functional, and behavioural factors in mouth breathing–related malocclusion.

The reduced maxillary width in MB children may result from disrupted intraoral pressure balance. Lack of tongue-palate contact weakens lateral expansion, allowing cheek pressure to constrict the arch, leading to a high-vaulted palate and reduced stability.^11^^,^^24^^,^^25^ Low tongue posture limits intrinsic muscle activity—especially the transverse fibres—affecting tongue shape, promoting malocclusion, and potentially contributing to orofacial discomfort.^26^

Resting tongue posture appears to exert a more sustained influence on maxillary development than transient functions such as swallowing or speech.

CBCT airway analysis revealed significant narrowing of the retropalatal and retroglossal regions in mouth-breathing children, likely attributable to posterior displacement of the soft palate and a lowered tongue posture. Additionally, decreased hyoid–mandibular distance and peripharyngeal muscular hypotonia may further compromise airway patency.^27^^,^^28^

These alterations may impair oxygenation and disrupt sleep, affecting growth and neurocognitive development.^4^^,^^8^ Concurrent maxillary constriction and reduced airway volume suggest a shared developmental disruption from altered orofacial function. CBCT facilitates the detection of subtle anatomical changes frequently overlooked in clinical examination, underscoring mouth breathing during growth as a modifiable risk factor for dentofacial and airway abnormalities.

If left unaddressed, persistent mouth breathing may predispose affected children to enduring craniofacial abnormalities and neurodevelopmental deficits, reinforcing the imperative for early, coordinated multidisciplinary intervention involving pediatricians, otolaryngologists, and orthodontists.^1^^,^^14^^,^^29^ Limitations of this study include its cross-sectional design, regional sample restriction, and potential selection bias, which limit causal inference and generalizability. Future longitudinal, multicenter studies are warranted to validate these findings and elucidate mechanistic pathways. Nevertheless, integrating CBCT into pediatric orthodontic evaluation protocols offers a promising strategy for timely identification and comprehensive management of skeletal and airway deviations, ultimately improving long-term functional and developmental outcomes.

## 5 Conclusion

1. Mouth-breathing children had significantly narrower maxillary widths than nasal breathers: MWM (60.93 mm vs. 67.66 mm), IMW (32.35 mm vs. 38.97 mm), MWC (34.91 mm vs. 44.70 mm), and ICW (21.99 mm vs. 30.13 mm) (p < 0.001).
2. Pharyngeal airway volumes were reduced in mouth breathers: NPV (6572.09 mm^3^ vs. 11,697.48 mm^3^) and OPV (24,342.17 mm^3^ vs. 68,005.19 mm^3^) (p < 0.001).
3. Cross-sectional airway areas were significantly smaller in the mouth-breathing group: NPA (100.92 mm^2^ vs. 194.06 mm^2^) and OPA (269.82 mm^2^ vs. 417.55 mm^2^) (p < 0.001).
4. These results highlight the impact of altered respiratory function on craniofacial and airway development, supporting early diagnosis and intervention.

## Patient's/guardian's consent statement

Written informed consent was obtained from all parents or guardians, along with assent from the children, ensuring full understanding and voluntary participation**.**

## Ethical clearance statement

Ethical approval was granted by the Institutional Review Board of Faculty of Dentistry, Universitas Gadjah Mada and RSGM UGM Prof. Soedomo (No. 232/UN1/KEP/FKG-RSGM/EC/2024). Parental consent and child assent were obtained prior to enrollment.

## Funding

The authors acknowledge the support of the Community Research Grant Fund of the Faculty of Dentistry, 10.13039/501100012521Universitas Gadjah Mada (No. 3918/UN1/KG/Set.KG1/LT/2024).

## Declaration of competing interest

The authors declare that they have no known competing financial interests or personal relationships that could have appeared to influence the work reported in this paper.
